# Efficacy of eleven antimicrobials against a gregarine parasite (Apicomplexa: Protozoa)

**DOI:** 10.1186/1476-0711-6-15

**Published:** 2007-11-12

**Authors:** Shajahan Johny, Amber Merisko, Douglas W Whitman

**Affiliations:** 1 Department of Biological Sciences, Box 4120, Illinois State University, Normal, Illinois, 61790, USA

## Abstract

**Background:**

The Apicomplexa are a diverse group of obligate protozoan parasites infesting a wide range of invertebrate and vertebrate hosts including humans. These parasites are notoriously difficult to control and many species continue to evolve resistance to commercial antibiotics. In this study, we sought to find an effective chemotherapeutic treatment against arthropod gregarines (Apicomplexa), and to identify candidate compounds for testing against other groups of protozoan parasites.

**Methods:**

We tested eleven commercial antibiotics against a gregarine parasite of *Romalea microptera *grasshoppers. Infected insects were fed daily, lettuce containing known amounts of specific antibiotics. On Days 15 or 20, we measured the number of gregarines remaining in the digestive tract of each grasshopper.

**Results:**

Treatment with metronidazole and griseofulvin in host insects significantly reduced gregarine counts, whereas, gregarine counts of insects fed, albendazole, ampicillin, chloramphenicol, fumagillin, quinine, streptomycin, sulfadimethoxine, thiabendazole or tetracycline, were not significantly different from the controls. However, albendazole produced a strong, but non-significant reduction in gregarine count, and streptomycin exhibited a non-significant antagonistic trend.

**Conclusion:**

Our results confirm that gregarine infections are difficult to control and suggest the possibility that streptomycin might aggravate gregarine infection. In addition, the insect system described here, provides a simple, inexpensive, and effective method for screening antibiotics.

## Background

The phylum Apicomplexa consists of unicellular protozoan parasites, infesting a wide range of Metazoa [1,2]. Included are numerous genera that attack humans or domesticated animals (e.g., *Plasmodium*, *Toxoplasma*, *Cryptosporidium*, *Neospora*, *Theileria*, *Babesia*, and *Eimeria*) [3,4]. In aggregate, these parasites cause great suffering and economic damage, and contribute to millions of human deaths each year [5]. Chemotherapeutic control ranges from non-existent to fairly effective; however, many species of Apicomplexa continue to evolve resistance to commercial antibiotics. For example, although most *Plasmodium *infections can still be cured by appropriate antimalarial drugs if treatment is administered early enough, resistance is increasing rapidly to essentially all compounds in use [5]. Clearly there is an urgent need to develop novel chemotherapeutic approaches against these diseases.

Gregarines (Apicomplexa of the subclass Gregarinia Dufour, 1828) are perhaps the most ubiquitous and taxonomically diverse of all parasites, infecting a wide range of invertebrate hosts, including arthropod vectors of vertebrate diseases [1,6-11]. Gregarines are considered to represent an early diverging apicomplexan lineage, thus making them a key group for questions regarding apicomplexan evolution [12]. Phylogenetic analysis of the small subunit (SSU) ribosomal RNA (rRNA) gene suggest that the gregarines are a sister group to the *Cryptosporidium*, a group parasitic on vertebrates [13-15]. Moreover, gregarines and *Cryptosporidium *share many life cycle features [16,17], and both groups lack a plastid genome, which is present in other apicomplexans [12,18]. Gregarines do not appear to directly impact human health. However, one gregarine species influences a human disease; the gregarine parasite *Ascogregarina culicis *apparently helps to maintain Chikungunya virus in vector mosquitoes [19], thereby fostering febrile epidemics in Southeast Asia and Africa [20-22], and recently spreading over Europe [23-25].

Although gregarines do not attack vertebrates, they have harmed, and continue to harm, scientific research, including research on arthropod vectors of human and animal diseases. This is because gregarines are extremely common in both field and laboratory arthropods [11,26,27] yet few researchers are aware of their presence or how these parasites may influence their experiments [10,28-30]. Gregarines are often considered to be sub-lethal or even harmless to their hosts, but in fact, they divert host nutrients to their own use, occupy space, alter host immune systems, and damage host cell walls when emerging, and thus foster microbial attack. As such, they can reduce longevity, vitality, or fecundity, or cause rapid mortality [27,31-38]. The effects of gregarines are seldom examined by researchers who study arthropod vectors of vertebrate diseases. Yet recent studies demonstrate that gregarine infection significantly increases the effectiveness of both chemical and microbial control measures against insect pest [28]. Hence, controlling an arthropod vector may hinge on the presence of gregarines.

Knowing the effects of gregarine parasites on their hosts requires a comparison of gregarine-infested and gregarine-free hosts. However, there are few effective antibiotic treatments to eliminate gregarines; typically, parasite numbers can be reduced, but seldom completely eliminated [39-41]. Sanitation and sterilization are commonly employed to combat gregarines, but these methods are sometimes laborious and ineffective [24,29]. The main problem with sterilization of host eggs and subsequent rearing is that it takes too long, especially for hosts that undergo only a single generation per year. Hence, there is a need to rapidly eliminate gregarines from hosts. Controlling other Apicomplexa is important as well. We are seeing increased levels of Apicomplexa infection in immune-repressed, HIV-infected patients, in part because of the evolution of drug resistance [5]. Numerous other human apicomplexan diseases like cryptosporidiosis are likewise difficult to treat [5,42]. Thus, there is a need to identify new antimicrobials for use against this group.

In this paper, we test 11 different commercial antibiotics for control of a gregarine (*Gregarina *sp.) in an insect, the Eastern Lubber Grasshopper, *Romalea microptera *(Beauvois). Our primary goal is to find an effective chemotherapeutic treatment against arthropod gregarines, and identify candidate compounds for testing against other related groups of apicomplexan parasites. Our secondary goal is to develop an inexpensive and effective insect-based system for rapidly testing large numbers of antibiotics *in vivo*.

## Methods

### Insect host

All experiments were conducted on Eastern Lubber grasshoppers, *Romalea microptera *obtained from stock colonies at Illinois State University [43]. The colony was established in 1997 from wild animals collected from Copeland, Florida, USA. A survey of the colony in 2003 indicated ~96% prevalence of gregarine infection. The origin of this infection is unknown.

### Gregarine parasite

**Figure 1:**
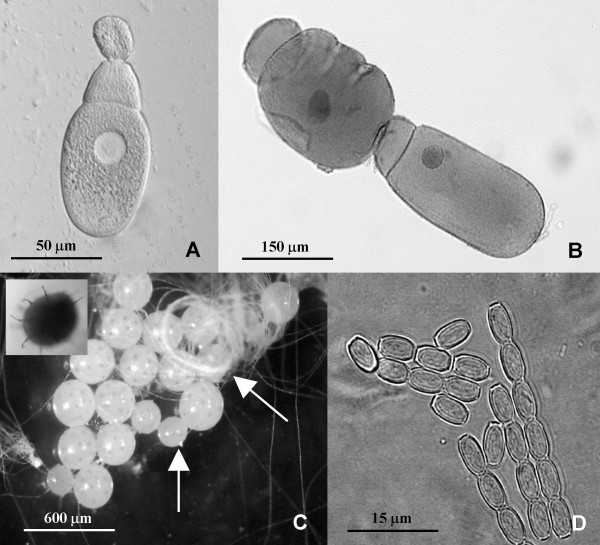
Life stages of *Gregarina *sp. infecting *Romalea microptera *grasshoppers. A. Fresh smear of trophozoite with epimerite; B. Gamonts on conjugation-stained with Heidenhain's iron haemotoxylin ; C. Gametocyst on sporulation (vertical arrow – unsporulated gametocyst, right arrow – coiled spores, inner picture – gametocyst showing sporoducts); D. Fresh spores

We used an unknown species of *Gregarina*. Placement into this genus was based on trophozoite with papillae shaped epimerite, early association, and gametocysts dehiscing through multiple sporoduct (Fig. 1A-D) [1,11] (Johny and Whitman, unpubl.). The natural occurrence and host range of this specific parasite is unknown. Indeed, probably more than 95% of the world's gregarine fauna awaits identification [11]. Gregarine gametocysts were collected from the faeces of captive male and female grasshoppers, washed with sterile distilled water 3–5 times, and incubated in air at 30°C under 90% RH [44], until dehiscence occurred. The resulting oocyst coils were disrupted by centrifugation in sterile distilled water at 1000 rpm for 5 minutes, resuspended, and then fed to grasshoppers. The oocyst concentration was determined using a haemocytometer, as per Undeen and Vavra [45].

### Compounds tested

We tested the following 11 commercially available products: albendazole, powder, 100% methyl 5-(propylthio)-2-benzimidazole carbonate (Sigma-Aldrich Inc., St. Louis); ampicillin, sodium salt, 100% monosodium D-(-)-6-(2-amino-2-phenylacetamido)-3,3-dimethyl-7-oxo-4-thia-1-azabicyclo [3.2.0]heptane-2-carboxylate (Research Products International, Mt. Prospect, IL); chloramphenicol, powder, 100% 2,2-dichloro-N-(2-hydroxy-1-[hydroxymethyl]-2-[4-nitrophenyl]ethyl)-, (R-[R*,R*]) (Research Products International); fumagilin-B (fumagillin), powder, 2.1% bicyclohexylammonium fumagillin (Medivet Pharmaceuticals Ltd., High River, Alberta, Canada); griseofulvin, powder, 95% (2S,6'R)-7-chloro-2',4,6-trimethoxy-6'-methyl-3H,4'H-spiro [1 benzofuran-2,1'-cyclohex[2]ene]-3,4'-dione (Sigma-Aldrich); metronidazole, powder, 100% (1-(beta-hydroxyethyl)-2-methyl-5-nitroimidazole (Sigma-Aldrich); quinine hemisulfate, salt, 94% (9*R*)-6'-methoxycinchonan-9-ol (Sigma-Aldrich); streptomycin sulfate, powder, 100% O-2-deoxy-2-(methylamino)-alpha-L-glucopyranosyl-(1-2)-O-5-deoxy-3-C-formyl-alpha-L-lyxofuranosyl-(1–4)-N,N'-bis-(aminoiminomethyl)-D-streptamine (Research Products International); sulfadimethoxine, powder, 100% 4-amino-N-[2,6-dimethoxy-4-pyrimidinyl]-benzenesulfonamide, (sodium salt) (Sigma-Aldrich); tetracycline HCl, powder, 100% 2-naphthacenecarboxamide, 4-(dimethylamino)-1,4,4a,5,5a,6,11,12a-octahydro-3,6,10,12,12a-pentahydroxy-6-methyl-1,11-dioxo-, (4S-[4alpha,4aalpha,5aalpha,6beta,12aalpha]) (Research Products International); and thiabendazole, powder, 99% 2-(4'-thiazolyl) benzimidazole (Sigma-Aldrich).

### Preparation of test compounds

We prepared oral doses of the various antibiotics by mixing them with juice from fresh carrot (*Daucus carota*) leaves. Carrot leaf juice is a strong phagostimulant for *R. microptera*. Juice was prepared without adding water, using a commercial juicer, and then filtered through cheesecloth. The water-soluble drugs were directly mixed with the filtrate. The hydrophobic drugs (albendazole, griseofulvin, metronidazole, and quinine) were first mixed with dry powdered sucrose, then blended into the pure juice, to produce suspensions. We then applied 20 μl of juice, containing a known concentration of drug onto 2 cm^2 ^pieces of fresh Romaine lettuce leaves, spreading the juice evenly over the surface. We allowed the juice to dry at air temperature (~10 min) and then fed one leaf to each grasshopper. All test compound mixtures were prepared immediately prior to feeding.

### Determination of proper doses for antibiotics

We prepared a wide range of concentrations of each antibiotic in carrot juice, as above, and then determined the maximum concentrations acceptable to adult male grasshoppers (the highest dose that 90% of hungry grasshoppers would consume). Once the maximum accepted dose was determined, we then selected our test doses as 50% of the maximum accepted dose (Table 1). Note that our purpose was not to compare identical doses of different antibiotics, but to identify a specific oral dose of antibiotic that was both palatable to grasshoppers and effective in eliminating gregarine infection in our test animal.

**Table 1:** Dosage for eleven antibiotics tested against a gregarine pathogen infecting *Romalea microptera *grasshoppers.

	**Mean dose per individual grasshopper**			
		
**Treatment group**	Dose offered (mg/day)	Dose consumed (mg/day) ± SE*	Cumulative dose consumed (mg/19 day) ± SE	**Mean dose (mg/gram fresh mass/day)**^†^
Control	None^‡^	None^‡^	None^‡^	None^‡^

Albendazole	1.00	0.84 ± 0.16	15.95 ± 1.24	0.23
Ampicillin	5.00	3.77 ± 1.03	71.65 ± 7.44	1.05
Chloramphenicol	5.52	4.19 ± 0.51	79.75 ± 4.96	1.17
Fumagilin-B	0.10	0.10 ± 0.00	1.94 ± 0.03	0.03
Griseofulvin	5.52	4.66 ± 1.01	88.66 ± 11.08	1.30
Metronidazole	0.10	0.06 ± 0.03	1.18 ± 0.25	0.02
Quinine	2.96	2.47 ± 0.34	47.02 ± 6.43	0.69
Streptomycin	5.00	4.59 ± 0.48	87.25 ± 3.50	1.28
Sulfadimethoxine	0.38	0.10 ± 0.03	1.88 ± 0.74	0.03
Tetracycline	5.26	4.73 ± 0.49	89.93 ± 4.69	1.32
Thiabendazole	0.68	0.53 ± 0.16	10.15 ± 1.18	0.15

*Not all individuals consumed a full dose each day.

^† ^[Mean dose consumed/animal/day]/average mass of a typical male

^‡^The Control group was given carrot leaf juice only.

### Treatment procedure

Ten male *Romalea microptera *(5- to 40-day-old adults) were tested with each drug. Grasshoppers were numbered individually with permanent markers and placed into individual, ventilated, 500-ml transparent plastic containers. Each day, for the next 19 days, each grasshopper was starved for12 hr, then fed a 2 cm^2 ^piece of Romaine lettuce smeared with 20 μl of juice + antibiotic. Insects were allowed to feed on the treated lettuce for 4 hr, and the amount of chemical ingested was calculated based on the amount of lettuce eaten by the insect. Note that not all insects consumed a full dose of antibiotic each day; hence, the dose consumed differs from the dose offered (Table 1). A control group of 40 insects received lettuce with pure carrot juice. After each day's antibiotic feeding, grasshoppers were fed *ad lib *Romaine lettuce and oatmeal for 8 hr, followed by the next 12 hr starvation period. Insects were maintained at 26 ± 2°C; 60–85% RH, and 14:10 (L:D) photoperiod. On Day 10, the control and treatment groups were inoculated with ~3000 gregarine oocysts/grasshopper by feeding each 1 cm^2 ^of Romaine lettuce contaminated with oocysts (collected as previously described). On Days 5 and 10, one or two grasshoppers from each treatment group and five from the control were dissected and examined for gregarines. These examinations looked for early and profound control; however, no dramatic effects were found in these early inspections, and therefore, these low-n results are not reported here. On Day 15, we dissected one or two grasshoppers from each treatment group, and on Day 20, all survivors were dissected and examined for gregarines.

### Evaluation of drug effect

To quantify the gregarines in individual insects, we removed and opened the host's digestive tract, under Yeager's physiological saline [46]. We examined the gut contents under a dissecting microscope, and counted the number of trophozoites, gamonts, and gametocysts. In this species, gamonts were always associated (conjugation), and such associated gamonts and gametocyst were counted as two individuals. Thus the total number of gregarines per grasshopper = # trophozoites + 2 × (# associated gamonts + # gametocysts). Our gregarine parasite requires more than 20 days to complete its life cycle at 26 ± 2°C. Hence, in our experiment, when we found individuals to be gregarine-free on days 15 or 20, we assumed that this had resulted from antibiotic treatment. We analyzed the combined data for Days 15 and 20 by one way ANOVA weighted for unequal variances, and the difference among treatment groups were identified using Tukey's honesty significant difference test. The data were normalized by log transformation before carrying out the ANOVA. All analysis were completed using Statistica 5.5 (StatSoft, Inc. Tulsa, OK, U.S.A) with α = 0.05.

## Results

**Figure 2:**
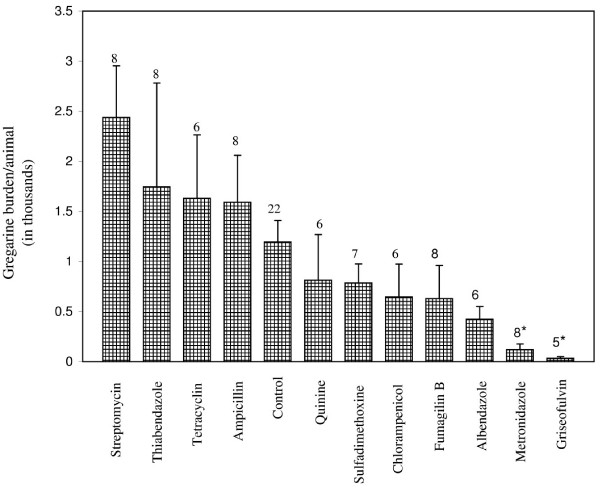
Mean ± SE number of gregarines in the guts of *Romalea microptera *grasshoppers (combination of animals dissected on Days 16 and 20) after treatment with different antimicrobials. Numbers above bar represents n. *Means significantly different from control group (ANOVA protected Tukey's HSD test, α = 0.05).

The mean densities of gregarines (pooled data for Days 15 and 20) for the different treatment groups are presented in Figure 2. Grasshoppers treated with metronidazole (ANOVA followed by Tukey HSD test, *P *< 0.01) and griseofulvin (ANOVA followed by Tukey HSD test, *P *< 0.05) exhibited significantly lower gregarine counts than control insects (Fig. 2). No other treatments were significantly different from the control; however, albendazole treatment exhibited a strong, but non-significant trend for reduced gregarine count (*P *> 0.05; Fig. 2). In contrast, a non-significant antagonistic trend was observed in the streptomycin treatment, which showed higher gregarine counts in comparison to the control (*P *> 0.05) (Fig. 2). Of the 98 animals dissected in Fig. 2, 1 control, 1 fumagillin-, 2 griseofulvin- and 4 metronidazole-treated animals lacked gregarines in their guts.

## Discussion

Of the 11 antimicrobials tested in this experiment, griseofulvin was the most effective in reducing gregarine parasites in the gut lumina of grasshoppers, followed by metronidazole. Griseofulvin, a chlorine-containing metabolite of the fungus *Penicillium griseofulvum *Direck [47], has been successfully used against onychomycosis (fungus), and used for more than three decades to treat dermatomycoses in humans and animals. It inhibits nucleic acid synthesis and its main effect on mitosis is due to disorganization of spindle microtubules in the M phase [48,49]. To our knowledge, griseofulvin has never been used to treat any apicomplexan parasite groups. Hence the potential use of griseofulvin against gregarines and related apicomplexan groups should be explored.

Metronidazole is a benzimidazole, known for its strong antiprotozoan activity, first used to treat *Trichomonas vaginalis *(Phylum: Zoomastigina) and later *Entamoeba histolytica *(Phylum: Sarcomastigophora), *Giardia lamblia *(Sarcomastigophora) and *Cryptosporidium *sp. (Phylum: Apicomplexa) [50-53]. It is administered in an inactive form and enters cells by passive diffusion [54]. The drug is then activated in cells via electron transfer from ferredoxin to its nitro group, resulting in the therapeutically active intermediate [55]. In the present study, metronidazole reduced gregarine mean intensities by 90% over controls, an effect comparable with the 71% reduction reported by Smith and Clopton [40] for treatment with metronidazole in their gregarine-cockroach model system. The dosage used in the present study (20 mg/kg body weight/day) is 50-fold higher than the dosage (0.4 mg/kg body weight/day) used by Smith and Clopton [40]. Moreover, their treatment regime was only for 5 days, whereas we administered metronidazole up to 19 days. However, on Day 10, we introduced a large dose of gregarine oocysts (~3000) into our test animals. Overall, our study reaffirms that metronidazole is a strong gregarinostat.

Albendazole, another benzimidazole with broad-spectrum antihelminthic and antifungal activity, is also effective against various protozoa [56]. It binds to tubulin and affects cytoskeletal microtubules; this property makes it potentially useful in the treatment of some protozoan infections in addition to its more established roles in therapy for helminthic infections. Albendazole is also strongly active against *Plasmodium berghei *and *P. falciparum *[57-59]. In our experiments, treatment with albendazole resulted in a strong, but non-significant decrease in the gregarine count. The lack of significance may be due to the poor absorption rate of albendazole across the gut. However, albendazole absorption is enhanced in mammals by taking it with fatty meals [60].

Earlier reports suggested that sulfadimethoxine displays some apicomplexan toxicity and this, and related compounds, are commonly used for prophylactic control of coccidians in livestock and poultry [1,61,62]. Recently Clopton and Smith [41] evaluated the activity of sulfadimethoxine against *Gregarina cubensis *and *Protomagalhaensia granulosae *(Apicomplexa: Eugregarinida), infecting the Death's Head cockroach, *Blaberus discoidalis*, and found that sulfadimethoxine significantly reduced the mean gregarine intensities, but did not eliminate the gregarine infection completely. In our test, sulfadimethoxine did not significantly reduce the gregarine numbers even after 19 days of treatment. One possible reason might be due to the dosage: the 30 mg/kg body weight used in our experiment is 5–6 times lower than the dosage (170 mg/kg body weight) used by Clopton and Smith [41] in their cockroach-gregarine model. Mourya et al. [30] assessed the efficacy of sulfadimethoxine against *Ascogregarina culicis *(Eugregarinida: Lecudinidae) infecting larval *Aedes aegypti *mosquitoes. Though high concentration of sulfadimethoxine (0.5 mg/ml culture water) reduced the mean gregarine intensity, it also resulted in host mortality of upto 52%.

In our study, streptomycin-treated animals had, on average, double the gregarine load of control animals. Although the difference was not significant, it suggests the possibility that streptomycin aggravated gregarine infection. In humans, gut microbiota effectively limit the capacity of invading micro-organisms, including pathogens, to colonize the gut, giving rise to what has been termed 'colonisation resistance' [63]. Streptomycin is a strong bacterial antibiotic which inhibits protein synthesis by binding to the 30S ribosomal subunit [64]. In the present study, streptomycin may have reduced midgut bacteria in grasshoppers, thereby increasing the survival of gregarines. Toure et al. [65] reported that combination antibiotics such as gentamicin, penicillin, and streptomycin reduced the bacterial flora in the mosquito midgut, which in turn increased the infectivity of *Plasmodium falciparum *in *Anopheles *mosquitoes. Likewise, *Culex bitaeniorhynchus *mosquitoes treated with tetracycline showed an increased susceptibility to the Japanese encephalitis (JE) virus [30].

## Conclusion

The gregarines (Apicomplexa) appear to be highly resistant to chemotherapy, although our results suggest that metronidazole and griseofulvin have moderate antibiotic effects. Streptomycin might aggravate gregarine infections. Apicomplexa parasites have a global impact on economic development and on the health and survival of millions of people and domesticated animals worldwide. New therapies for the diseases they cause are urgently required, but many drugs have proven ineffective [42] or are becoming increasingly ineffective because of increased antibiotic resistance in pathogens [66]. The insect system described here is a simple method for evaluation of drugs against gregarines. It can also serve as a model system to identify compounds with a potential broad activity against other Apicomplexa. Insects can be cheaply mass-reared, require less laboratory space and support facilities, do not suffer from the ethical problems of using mammals as experimental hosts, and share some common immune-response features with vertebrates [67]. Insects can be infected by virtually all pathogen and parasite taxa, including viruses, rickettsiae, bacteria, fungi, protozoa, and nematodes, etc. Therefore, insects can be exploited, not only to examine pathogens of arthropods, but also to screen new drugs for potential use against a wide range of pathogens and parasites of vertebrates [40,41,68]. Finally, the effects of gregarines on arthropod vectors of vertebrate diseases have been largely ignored. Understanding such effects will require the ability to culture gregarine-free hosts. This research contributes to that goal.

## Authors' contributions

SJ and AM performed experimental work. DWW and SJ designed the study, collected and analyzed the data and drafted the manuscript. All authors read and approved the final manuscript.

## References

[B1] Levine N (1988). The Protozoan Phylum Apicomplexa.

[B2] Ellis J, Morrison DA, Jeffries AC, Coombs G, Vickerman K, Sleigh M, Warren A (1989). The phylum Apicomplexa: an update on the molecular phylogeny. Evolutionary Relationships Among Protozoa.

[B3] O'Donoghue PJ (1995). *Cryptosporidium *and cryptosporidiosis in man and animals. Int J Parasitol.

[B4] Kim K, Weiss LM (2004). *Toxoplasma gondii*: the model apicomplexan. Int J Parasitol.

[B5] Hammarton TC, Mottram JC, Doerig C (2003). The cell cycle of parasitic protozoa: potential for chemotherapeutic exploitation. Prog Cell Cycle Res.

[B6] Levine ND (1977). Revision and checklist of the species (other than lecudina) of the aseptate gregarine family Lecudinidae. J Protozool.

[B7] Warburg A, Ostrovska K (1991). Host-parasite relationships of *Ascogregarina chagasi *(Eugregarinorida, Aseptatorina, Lecudinidae) in *Lutzomyia longipalpis *(Diptera: Psychodidae). Int J Parasitol.

[B8] Ostrovska K, Warburg A, Montoya-Lerma J (1990). *Ascogregarina saraviae *n. sp. (Apicomplexa: Lecudinidae) in *Lutzomyia lichyi *(Diptera: Psychodidae). J Protozool.

[B9] Beier JC (1983). Effects of gregarine parasites on the development of *Dirofilaria immitis *in *Aedes triseriatus *(Diptera: Culicidae). J Med Entomol.

[B10] Mourya DT, Soman RS (1985). Effect of gregarine parasite, *Ascogregarina culicis *& tetracycline on the susceptibility of *Culex bitaeniorhynchus *to JE virus. Indian J Med Res.

[B11] Clopton R, Lee JJ, Leedale G, Patterson D, Bradbury PC (2002). Phylum Apicomplexa Levine, 1970: Order Eugregarinorida Leeger, 1900. Illustrated Guide to the Protozoa.

[B12] Toso MA, Omoto CK (2007). *Gregarina niphandrodes *may lack both a plastid genome and organelle. J Eukaryot Microbiol.

[B13] Carreno RA, Martin DS, Barta JR (1999). *Cryptosporidium *is more closely related to the gregarines than to coccidia as shown by phylogenetic analysis of apicomplexan parasites inferred using small-subunit ribosomal RNA gene sequences. Parasitol Res.

[B14] Leander B, Clopton RE, Keeling PJ (2003). Phylogeny of gregarines (Apicomplexa) as inferred from small-subunit rDNA and -tubulin. Int J Syst Evol Microbiol.

[B15] Xiao L, Fayer R, Ryan U, Upton SJ (2004). *Cryptosporidium *taxonomy: recent advances and implications for public health. Clin Microbiol Rev.

[B16] Hijjawi NS, Meloni BP, Ryan UM, Olson ME, Thompson RC (2002). Successful in vitro cultivation of *Cryptosporidium andersoni*: evidence for the existence of novel extracellular stages in the life cycle and implications for the classification of *Cryptosporidium*. Int J Parasitol.

[B17] Rosales MJ, Cordon GP, Moreno MS, Sanchez CM (2005). Extracellular like-gregarine stages of *Cryptosporidium parvum*. Acta Trop.

[B18] Abrahamsen MS, Templeton TJ, Enomoto S, Abrahante JE, Zhu G, Lancto CA, Deng M, Liu C, Widmer G, Tzipori S (2004). Complete genome sequence of the apicomplexan, *Cryptosporidium parvum*. Science.

[B19] Mourya DT, Singh DK, Yadav P, Gokhale MD, Barde PV, Narayan NB, Thakare JP, Mishra AC, Shouche YS (2003). Role of gregarine parasite *Ascogregarina culicis *(Apicomplexa: Lecudinidae) in the maintenance of Chikungunya virus in vector mosquito. J Eukaryot Microbiol.

[B20] Rao T (1966). Recent epidemics caused by Chikungunya virus in India 1963–1965. Scientific Culture.

[B21] Powers A, Brault AC, Tesh RB, Weaver SC (2000). Reemergence of Chikunggunya and o'nyong-nyong viruses: evidence of distinct geographical lineages and distant evolutionary relationships. J Gen Virol.

[B22] Jupp P, McIntosh BM, Monath TP (1988). Chikungunya virus disease. Arbovirus: Epidemiology and Ecology.

[B23] Savarino A, Cauda R, Cassone A (2007). On the use of chloroquine for chikungunya. Lancet Infect Dis.

[B24] Lines J (2007). Chikungunya in Italy. BMJ.

[B25] Watson R (2007). Europe witnesses first local transmission of chikungunya fever in Italy. BMJ.

[B26] Johny S, Muthukumaravel K, Raghu S, Muralirangan MC, Sanjayan KP (1999). Geographical distribution of cephaline gregarines (Apicomplexa: Protozoa) in relation to grasshoppers (Orthoptera: Acrididae) in Tamil Nadu, India. Int J Ecol Environ Sci.

[B27] Pushkala K, Muralirangan MC (1997). Impact of *Gregarina subramanii*, a new gregarine species on the biology of the grasshopper; *Eyprepocnemis alacris *alacris (Serville). The Entomologist.

[B28] Lopes RB, Alves SB (2005). Effect of *Gregarina *sp. parasitism on the susceptibility of *Blattella germanica *to some control agents. J Invertebr Pathol.

[B29] Dougherty MJ, Ward RD (1991). Methods of reducing *Ascogregarina chagasi parasitaemia *in laboratory colonies of *Lutzomyia longipalpis*. Parassitologia.

[B30] Mourya D, Mahadev PVM, Dhanda V (1985). Effect of antiamoebic drugs on *Ascogregarina culicis*, gregarine parasite of *Aedes aegypti*. Indian Journal of Parasitology.

[B31] Fukuda T, Willis OR, Barnard DR (1997). Parasites of the Asian tiger mosquito and other container-inhabiting mosquitoes (Diptera:Culicidae) in northcentral Florida. J Med Entomol.

[B32] Ghose S, Sengupta T, Halder DP (1985). Two new septate gregarine (Apicomplexa: sporozoea), *Gregarina basiconstrictonea *n. sp. and *Hirmocystis oxeata *n. sp. from *Tribolium castaneum*. Acta Protozool.

[B33] Janardanan K, Ramachandran P (1982). Studies on a new cephaline gregarins, *Stenoductus trigoniuli *sp. n. with a note on its cytopathology. Arch Protistenkd.

[B34] Abro A (1974). The gregarine infection in different species of Odonata from the same habitat. Zool Scripta.

[B35] Purrini K, Keil H (1989). *Ascogregarina bostrichidorum *n. sp. (Lecudinidae, Eugregarinida), a new gregarine parasitizing the larger grain borer, *Prostephanus truncatus *Horn (1878) (Bostrichidae, Coleoptera). Arch Protistenkd.

[B36] Garcia JJ, Fukuda T, Becnel JJ (1994). Seasonality, prevalence and pathogenicity of the gregarine *Ascogregarina taiwanensis *(Apicomplexa: Lecudinidae) in mosquitoes from Florida. J Am Mosq Control Assoc.

[B37] Johny S, Muralirangan MC, Sanjayan KP (2000). Parasitization potential of two cephalogregarines, *Leidyana subramanii *Pushkala and Muralirangan and *Retractocephalus dhawanii *sp. nov. on the tobacco grasshopper *Atractomorpha crenulata *(Fab.). J Orthopt Res.

[B38] Ball S, Cunningham AA, Clarke D, Daszak P (1995). Septate gregarines associated with a disease of the hissing cockroach *Gromphadorhina portentosa*. J Invertebr Pathol.

[B39] Fajer-Avila E, Covarrubias MSM, Abad-Rosales S, Roque A, Meza-Bojorquez P, Meza-Bojorquez P, Hernandez-Gonzalez C (2005). Effectiveness of oral ElancobanTM and Avimix-STTM against *Nematopsis *(Apicomplexa: Porosporidae) gametocysts infecting the shrimp *Litopenaeus vannamei*. Aquaculture.

[B40] Smith A, Clopton RE (2003). Efficiency of oral metronidazole and potassium sorbate against two gregarine parasites, *Protomagalhaensia *granulose and *Gregarina cubensis *(Apicomlexa: Eugregarinida), infecting the death's head cockroach, *Blaberus discoidalis*. Comp Parasitol.

[B41] Clopton RE, Smith A (2002). Efficacy of oral sulfadimethoxine against two gregarine parasites, *Protomagalhaensia granulosae *and *Gregarina cubensis *(Apicomplexa: Eugregarinida), infecting the Death's Head cockroach, *Blaberus discoidalis*. J Parasitol.

[B42] Roberts CW, McLeod R, Rice DW, Ginger M, Chance ML, Goad LJ (2003). Fatty acid and sterol metabolism: potential antimicrobial targets in apicomplexan and trypanosomatid parasitic protozoa. Mol Biochem Parasitol.

[B43] Matuszek J, Whitman DW (2001). Captive rearing of eastern lubber grasshopper *Romalea microptera*. Proceedings of the Invertebrates in Captivity Conference, 2001.

[B44] Winston P, Bates DH (1960). Saturated solutions for the control of humidity in biological research. Ecology.

[B45] Undeen A, Vavra J, Lacey LA (1997). Research methods for entomopathogenic protozoa. Manual of techniques in insect pathology.

[B46] Yeager J (1939). Electrical stimulation of isolated heart preparations from *Periplaneta americana*. J Agric Research.

[B47] Oxford A, Raistruck H, Simonarat P (1939). Studies in the biochemistry of microorganisms IX. Griseofulvin CHOCI, a metabolic product of *Penicillium griseofulvin *Direck. Biochem J.

[B48] DeCarli L, Larizza L (1988). Griseofulvin. Mutat Res.

[B49] Finkelstein E, Amichai B, Grunwald MH (1996). Griseofulvin and its uses. Int J Antimicro Ag.

[B50] Conteas CN, Berlin OG, Ash LR, Pruthi JS (2000). Therapy for human gastrointestinal microsporidiosis. Am J Trop Med Hyg.

[B51] Savioli L, Smith H, Thompson A (2006). *Giardia *and *Cryptosporidium *join the 'Neglected Diseases Initiative'. Trends Parasitol.

[B52] Petri WA (2003). Therapy of intestinal protozoa. Trends Parasitol.

[B53] Xiao L, Herd RP, Rings DM (1993). Concurrent infections of *Giardia *and *Cryptosporidium *on two Ohio farms with calf diarrhea. Vet Parasitol.

[B54] Boreham PF, Phillips RE, Shepherd RW (1985). A comparison of the in-vitro activity of some 5-nitroimidazoles and other compounds against *Giardia intestinalis*. J Antimicrob Chemother.

[B55] Johnson P (1993). Metronidazole and drug resistance. Parasitol Today.

[B56] Liu L, Weller PF (1996). Antiparasitic drugs. N Engl J Med.

[B57] Dow GS, O'Hara AJ, Newton SC, Reynoldson JA, Thompson RC (2000). *Plasmodium berghei*: the antimalarial activity of albendazole in rats is mediated via effects on the hematopoietic system. Exp Parasitol.

[B58] Dow GS, Reynoldson JA, Thompson RC (1998). *Plasmodium berghei*: in vivo efficacy of albendazole in different rodent models. Exp Parasitol.

[B59] Skinner-Adams TS, Davis TM, Manning LS, Johnston WA (1997). The efficacy of benzimidazole drugs against *Plasmodium falciparum *in vitro. Trans R Soc Trop Med Hyg.

[B60] Awadzi K, Hero M, Opoku NO, Buttner DW, Coventry PA, Prime MA, Orme ML, Edwards G (1994). The chemotherapy of onchocerciasis XVII. A clinical evaluation of albendazole in patients with onchocerciasis; effects of food and pretreatment with ivermectin on drug response and pharmacokinetics. Trop Med Parasitol.

[B61] Cates L, Verderame M (1986). Sulfa drugs. Handbook of chemotherapeutic agents.

[B62] Chambers H, Jawetz E, Katzung BG (1998). Sulfonamides, trimethropin, and quinolones. Basic and clinical pharmacology.

[B63] Hentges D, Fuller R (1992). Gut flora and disease resistance. Probiotics: The Scientific Basis.

[B64] Sambrook J, Russel DW (2001). Molecular cloning, a laboratory manual.

[B65] Toure AM, Mackey AJ, Wang ZX, Beier JC (2000). Bactericidal effects of sugar-fed antibiotics on resident midgut bacteria of newly emerged anopheline mosquitoes (Diptera: Culicidae). J Med Entomol.

[B66] AKOVA M (2006). Emerging problem pathogens: A review of resistance patterns over time. International Journal of Infectious Diseases.

[B67] Salzet M (2001). Vertebrate innate immunity resembles a mosaic of invertebrate immune responses. Trends Immunol.

[B68] Guhl W (1999). The possibility of testing antiparasitical substances using the cockroach as a model. Parasitol Res.

